# Nova Mutação no Gene DSP – Um Caso de Cardiomiopatia Arritmogênica com Fenótipo Isolado do Ventrículo Esquerdo e Alto Risco de Morte Súbita

**DOI:** 10.36660/abc.20201087

**Published:** 2021-07-14

**Authors:** Pedro von Hafe Leite, Olga Azevedo, Geraldo Dias, Filipa Cardoso, Tamara Pereira, António Lourenço

**Affiliations:** 1 Hospital Senhora da Oliveira Departamento de Cardiologia Guimarães Portugal Hospital Senhora da Oliveira - Departamento de Cardiologia, Guimarães - Portugal; 2 Universidade do Minho Departamento de Cardiologia Braga Portugal Universidade do Minho - Departamento de Cardiologia, Braga - Portugal

**Keywords:** Morte Súbita Cardíaca, Doenças Cardiovasculares

## Introdução

A morte súbita cardíaca (MSC) em adultos jovens (18–35 anos) é causada mais frequentemente de cardiomiopatias hereditárias previamente não diagnosticadas. As causas mais comuns de MSC são a cardiomiopatia hipertrófica e a cardiomiopatia arritmogênica (CMA), seguidas de anomalias congênitas das artérias coronárias, miocardite, ruptura aórtica na síndrome de Marfan, defeitos de condução, e doenças valvulares.^1^

A CMA é responsável por até 20% dos casos de MSC em indivíduos abaixo dos 35 anos de idade.^2^ Em uma série de 86 vítimas jovens de MSC, a CMA representou 10,3% dos casos, sendo a segunda maior causa de MSC.^3^ A cardiomiopatia dilatada (CMD) é uma causa menos frequente de MSC entre jovens, representando aproximadamente 2% dos casos em atletas.^4^

A CMA é uma doença hereditária do músculo cardíaco que resulta em infiltração fibrogordurosa do miocárdio ventricular.^5^

A CMA é uma cardiomiopatia geneticamente determinada causada por mutações em genes codificantes de proteínas de desmossomos, que são estruturas intercelulares especializadas.^6^

A classificação atual da CMA inclui cardiomiopatia arritmogênica do ventrículo direito, variantes da doença biventricular, envolvimento predominante do ventrículo esquerdo (VE), e o fenótipo do VE caracterizado pelo envolvimento isolado do VE.^7^

O diagnóstico da CMA é baseado nos critérios do *Task Force Criteria* (TFC) modificados de 2010.^8^ Entretanto, esses critérios TFC modificados carecem de sensibilidade no diagnóstico da CMA com envolvimento do VE isolado ou predominante. Além disso, o diagnóstico diferencial da CMA de outras entidades, tais como CMD, sarcoidose ou miocardite, pode representar um desafio.

## Caso clínico

**Figura 1:**
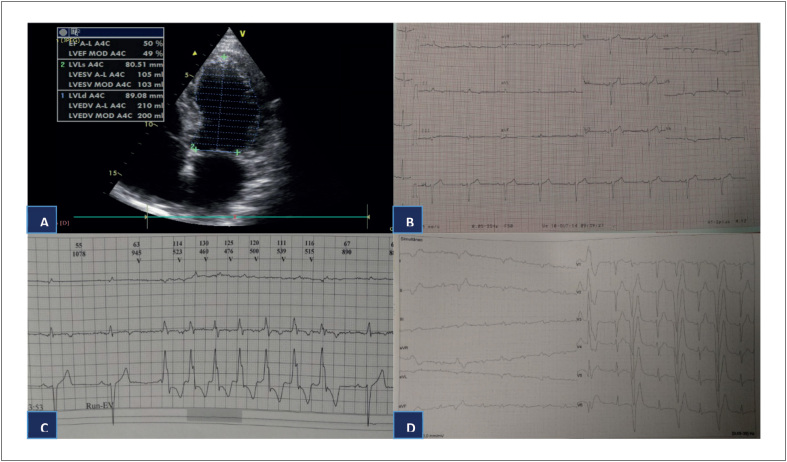
A) Ecocardiograma transtorácico (visão apical de quatro câmaras) mostrando dilatação e disfunção leve do ventrículo esquerdo. B) ECG mostrando o ritmo sinusal com avanço fraco da onda R em V1-V3, e ondas T negativas nas derivações I, II, III, aVF, aVL e V4-V6. C) Monitoramento por Holter durante 24 horas revelando TV não sustentada com sete bloqueios de ramos de feixe direito complexos e incompletos. D) Teste de stress por esforço demonstrando ectopia ventricular frequente, principalmente com origem no VE.

Um homem de 49 anos, com histórico de consumo de álcool leve a moderado, foi acompanhado em uma consulta cardiológica por 12 anos, com diagnóstico de CMD presumidamente causado pelo consumo de álcool. O ecocardiograma transtorácico mostrou dilatação leve das quatro câmaras e disfunção sistólica do VE leve com hipocinesia global (Figura 1A). O eletrocardiograma mostrou o ritmo sinusal com avanço fraco da onda R em V1-V3, e ondas T negativas nas derivações I, II, III, aVF, aVL e V4-V6 (Figura 1B). A cintilografia com perfusão do miocárdio resultou negativa para isquemia. O monitoramento pelo Holter de 24 horas mostrou o ritmo sinusal, aproximadamente 6000 batimentos ectópicos ventriculares multifocais uma TV não sustentada com sete bloqueios de ramos de feixe direito (BRFD) complexos e incompletos (Figura 1C). O teste de stress por esforço demonstrou ectopia ventricular frequente, principalmente com origem no VE (Figura 1D).

Depois de 12 anos de monitoramento, o paciente sofreu uma pré-síncope no trabalho, sendo levado imediatamente ao hospital pela equipe de emergência médica. Ao chegar ao hospital, o paciente desenvolveu fibrilação ventricular, e, apesar das medidas avançadas de suporte vital, acabou morrendo.

Dois dias depois de sua morte, seu filho de 16 anos, sem histórico patológico conhecido, foi encontrado desacordado por sua mãe, em sua cama enquanto dormia. O suporte vital foi iniciado assim que o serviço de emergência chegou, mas não foi bem-sucedido, e o adolescente morreu.

A esposa, a filha e sete irmãos do paciente zero foram submetidos a testagens com ECG, ecocardiograma, e Holter de 24 horas, todos os quais obtiveram resultados normais.

A autópsia do paciente zero revelou um coração aumentado, pesando 600 g, e aterosclerose coronária discreta. Não foram encontradas lesões isquêmicas agudas ou crônicas no exame macroscópico. Com base nesses achados, o relatório da autópsia concluiu que uma causa de morte por arritmia não poderia ser descartada. A autópsia do filho do paciente zero também revelou um coração aumentado, pesando 535g. O relatório da autópsia descreveu que o terço externo da circunferência da parede do VE parecia estar separado, em toda sua extensão longitudinal, dos dois-terços internos da parede do VE. Infelizmente, os relatórios histológicos não foram disponibilizados aos irmãos ou seus médicos.

O estudo genético post mortem revelou, em ambos os casos, a variante em heterozigose c.1080G>A (p.Trp360*) no gene *DSP*, classificada como provavelmente patogênica; e a variante c.3010G>T (p.Ala1004Ser) no gene *MYH6*, classificada como variante genética de significância incerta (GVUS).

**Figura 2:**
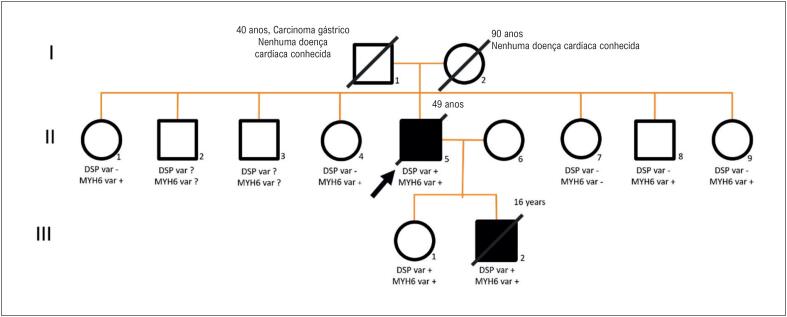
Linhagem familiar mostrando indivíduos afetados com CMA (símbolos escuros) e indivíduos não afetados (símbolos brancos). A seta indica o probando. A variante DSP está presente (+) em indivíduos afetados, estando ausentes (-) nos indivíduos não afetados. A variante MYH6 está presente (+) em indivíduos afetados, estando ausentes (-) nos indivíduos não afetados. DSP var: variante em heterozigose c.1080G>A (p.Trp360*) no gene DSP, classificada como provavelmente patogênica; MYH6 var: variante c.3010G>T (p.Ala1004Ser) no gene MYH6, classificada como variante genética de significância incerta.

Até agora, não se identificou nenhum paciente portador da variante *DSP*. A figura 2 mostra a linhagem familiar com os achados genéticos.

## Discussão

O diagnóstico da CMA é desafiador devido à ausência de critérios diagnósticos exclusivos e específicos, sua expressividade variável, e sua penetrância incompleta em familiares.^9^ A CMA, que foi inicialmente descrita como uma doença de VD isolada ou predominante, frequentemente apresenta envolvimento do VE, que pode estar presente ou até mesmo predominante nos primeiros estágios de alguns transportadores de mutação, expandindo o espectro clínico da doença.^9^

De acordo com o TFC modificado de 2010, o paciente zero apresentou um critério principal (identificação de mutação patogênica categorizada como associada ou provavelmente associada à CMA) e dois critérios menores (ondas T invertidas em V4-V6 e >500 batimentos ventriculares prematuros no monitoramento por Holter de 24 horas), que permitiram o diagnóstico definitivo da CMA.^8^

No entanto, como o TFC modificado careceu de sensibilidade no diagnóstico da CMA com envolvimento isolado ou predominante do VE, Corrado et al. recentemente apresentaram um documento de Consenso de Especialistas Internacionais propondo o “critério de Padua”, que constitui um aperfeiçoamento dos critérios diagnósticos de CMA com objetivo de diagnosticar todo o espectro das variantes fenotípicas da CMA.^10^

Nesse consenso recente, novos critérios foram acrescentados refletindo o envolvimento do VE, propondo que: (i) a disfunção sistólica do VE seja um critério menor para diagnóstico das variantes “biventricular” ou de “dominância esquerda” da doença; (ii) o realce tardio pelo gadolínio (RTG)/fibrose miocárdica do VE na forma de estria (ou faixa) afetando ≥1 segmentos da parede livre do VE, septo ou ambos seja um critério importante; (iii) anormalidades de repolarização com ondas T invertidas nas derivações precordiais esquerdas (V4-V6) (na ausência de BRFE completos) sejam um critério menor; (iv) anormalidades de despolarização com baixas tensões de QRS nas derivações do membro (na ausência de obesidade, enfisema, ou efusão pericárdica) sejam um critério menor, baseado na noção de que uma redução da massa miocárdica do VE por troca fibrogordurosa pode levar a baixas tensões de QRS; (v) Extrassístoles ventriculares frequentes (>500 em 24 horas), taquicardia ventricular não sustentada ou sustentada como morfologia de BRFD (exceto o padrão fascicular) sejam um critério menor; e (vi) a demonstração de uma mutação patogênica em genes relacionados à CMA sejam considerados um critério necessário para o diagnóstico em pacientes com CMA dominante esquerda e sem envolvimento do VD clinicamente detectável, porque é o achado mais específico ligando as características fenotípicas do VE à CMA.^10^

Realmente, todos os critérios mencionados são atendidos no paciente zero deste relatório (exceto mudanças nas IRM, já que não foram realizadas durante o acompanhamento), confirmando, portanto, o diagnóstico do CMA de dominância esquerda.

A CMD é particularmente difícil de distinguir das formas não clássicas de CMA. Essas duas entidades podem se sobrepor significativamente, que podem resultar em uma categorização incorreta do diagnóstico, como ocorreu provavelmente em nosso paciente zero. Mutações nos genes desmossômicos são relativamente comuns em pacientes com diagnóstico clínico de CMD, e são encontradas mutações em *DSP* em 3% dos pacientes com CMD.^11^

A CMA com dominância esquerda pode se apresentar, em uma faixa etária ampla, geralmente com palpitações e perda de consciência. A arritmia ventricular (AV) com morfologia de BRFD é característica e geralmente fora de proporção em relação ao grau de disfunção do VE.

Palpitações, (pré-)síncope e VA estão presentes em um estágio inicial da CMA, frequentemente na ausência de grandes anormalidades estruturais, conforme observado no paciente zero e em seu filho.

Estudo de genótipo/fenótipo sugeriram que mutações de DSP são associadas com um fenótipo grave com risco mais alto de AV e MSC, e um nível alto de envolvimento do VE, particularmente em pacientes com mutações, conforme observado nos pacientes deste estudo. Além disso, alinhado ao caso deste estudo, as mutações do *DSP* podem ser associadas à inversão da onda T nas derivações V4 a V6.^12^

No caso deste estudo, a variante p.Trp360* foi encontrada no gene *DSP*. Embora nunca tenha sido descrito na literatura ou em bancos de dados genéticos, essa mutação resulta em uma proteína truncada, que podem se relacionar a um fenótipo mais agressivo, como visto na família reportada neste estudo. Além disso, a análise de linhagem demonstrou um padrão de congregação positivo, já que a mutação do DSP foi identificada apenas nos pacientes afetados, e não nos pacientes com fenótipo negativo, inclusive os idosos.

Este caso mostra a importância do estudo genético *post-mortem* em pacientes com fenótipo de CMD/CMA, que sofreram MSC antes de que a testagem genética tivesse sido realizada.
