# Supplementary material for: Nova Mutação no Gene DSP – Um Caso de Cardiomiopatia Arritmogênica com Fenótipo Isolado do Ventrículo Esquerdo e Alto Risco de Morte Súbita
Source: Arq Bras Cardiol. 2021 Jul 14;117(1 Supl 1):29–32. [Article in Portuguese] doi: 10.36660/abc.20201087 (PMC8291893; doi:10.36660/abc.20201087)
Supplement: Translation

# Translation: Novel Mutation in *DSP* Gene – A Case of Arrhythmogenic Cardiomyopathy with Isolated Left Ventricular Phenotype and High Risk of Sudden Cardiac Death

**Authors:** Pedro von Hafe Leite, Olga Azevedo, Geraldo Dias, Filipa Cardoso, Tamara Pereira, António Lourenço

**Affiliations:** 1 Guimarães Portugal Hospital Senhora da Oliveira - Departamento de Cardiologia, Guimarães - Portugal; 2 Braga Portugal Universidade do Minho - Departamento de Cardiologia, Braga - Portugal

**Keywords:** Cardiac Sudden Death, Cardiovascular Diseases

## Introduction

Sudden cardiac death (SCD) in young adults (18–35 years) most commonly results from previously undiagnosed inherited cardiomyopathies. The most common causes of sudden cardiac death are hypertrophic cardiomyopathy and arrhythmogenic cardiomyopathy (ACM), followed by congenital anomalies of coronary arteries, myocarditis, aortic rupture in Marfan's syndrome, conduction defects, and valve diseases.^1^

ACM accounts for up to 20% of sudden cardiac death in individuals under 35 years of age.^2^ In a series of 86 victims of SCD at a young age, ACM accounted for 10.3% of the cases, being the second major cause of SCD.^3^ Dilated cardiomyopathy (DCM) is a less frequent cause of SCD in young individuals, accounting for nearly 2% of cases in athletes.^4^

ACM is an inherited heart muscle disorder that results from fibrofatty infiltration of the ventricular myocardium.^5^

ACM is a genetically determined cardiomyopathy caused by mutations in genes encoding proteins of desmosomes, which are specialized intercellular structures.^6^

The current classification of ACM includes the classic arrhythmogenic right ventricular cardiomyopathy, biventricular disease variants, predominant left ventricular (LV) involvement, and the LV phenotype characterized by isolated LV involvement.^7^

The diagnosis of ACM is based on the modified Task Force Criteria (TFC) from 2010.^8^ However, these modified TFC lack sensitivity in the diagnosis of ACM with isolated or predominant LV involvement. Furthermore, differential diagnosis of ACM from other entities, such as DCM, sarcoidosis or myocarditis, may be challenging.

## Clinical case

A 49-year-old man, with a history of mild to moderate alcohol consumption, was followed up in a cardiology consultation for 12 years with the diagnosis of DCM, presumably due to alcohol consumption. Transthoracic echocardiogram showed mild dilatation of four chambers and mild left ventricular systolic dysfunction with global hypokinesia (Figure 1A). The electrocardiogram revealed sinus rhythm with a poor progression of the R wave in V1-V3 and negative T wave in leads I, II, III, aVF, aVL, and V4-V6 (Figure 1B). Myocardial perfusion scintigraphy was negative for ischemia. A 24h-Holter monitoring showed a sinus rhythm, nearly 6,000 multifocal ventricular ectopic beats, and one non-sustained VT with seven complexes and incomplete right bundle branch block (RBBB) (Figure 1C). The exercise stress test showed frequent ventricular ectopy, mostly with LV origin (Figure 1D).

**Figure 1:** A) Transthoracic echocardiogram (apical four-chamber view) showing mild left ventricular dysfunction and dilatation. B) ECG showing sinus rhythm with poor progression of R wave in V1-V3 and negative T waves in leads I, II, III, aVF, aVL, and V4-V6. C) 24h-Holter monitoring revealing non-sustained VT with seven complexes and incomplete right bundle branch block. D) Exercise stress test, showing frequent ventricular ectopy, mostly with LV origin.

After 12 years of follow-up, the patient suffered a pre-syncope while at work, and was immediately taken to the hospital by the emergency medical team. Upon arrival at the hospital, the patient developed ventricular fibrillation and, despite the advanced life support measures, eventually died.

Two weeks after his death, his 16-year-old son, with no known pathological history, was found inanimate by his mother in his bed where he was sleeping. Advanced life support was initiated at the arrival of the emergency medical services, but it was unsuccessful and the teenager died.

Spouse, daughter, and seven siblings of the index case were submitted to screening with ECG, echocardiogram, and 24h-Holter, all of whom had normal results.

The autopsy of the index case showed an enlarged heart weighing 600g and discrete coronary atherosclerosis. No acute or chronic ischemic lesions were found on the macroscopic examination. Based on these findings, the autopsy report concluded that an arrhythmic cause of death could not be excluded. The autopsy of the son of the index case also showed an enlarged heart weighing 535g. The autopsy report described that the external third of the LV circumferential wall appeared to be detached, in all its longitudinal length, from the two inner thirds of the LV wall. Unfortunately, the histological reports were not made available for siblings or their physicians.

*Post-mortem* genetic study revealed, in both cases, the variant in heterozygosity c.1080G>A (p.Trp360*) in the *DSP* gene, classified as probably pathogenic, and the variant c.3010G>T (p.Ala1004Ser) in the *MYH6* gene, classified as a genetic variant of uncertain significance (GVUS).

So far, no relatives were found carrying the *DSP* variant. Figure 2 shows the family pedigree with the genetic findings.

**Figure 2:** Family pedigree showing affected individuals with ACM (dark symbols) and non-affected individuals (white symbols). The arrow indicates the proband. The DSP variant is present (+) in affected individuals, being absent (-) in non-affected ones. The MYH6 variant is present (+) in affected patients, but also in some non-affected relatives. DSP var: variant in heterozygosity c.1080G>A (p.Trp360*) in the DSP gene, classified as probably pathogenic; MYH6 var: variant c.3010G>T (p.Ala1004Ser) in the MYH6 gene, classified as a genetic variant of uncertain significance.

## Discussion

The diagnosis of ACM is challenging because of the absence of specific unique diagnostic criteria, its variable expressivity, and its incomplete penetrance in relatives.^9^ ACM, which was initially described as an isolated or predominant RV disease, exhibits frequent LV involvement, which may be present or even predominant at early stages in some mutation carriers, expanding the clinical spectrum of the disease.^9^

According to the modified TFC of 2010, the index case presented a major criterion (identification of pathogenic mutation categorized as associated or probably associated with ACM) and two minor criteria (inverted T waves in V4-V6 and > 500 ventricular premature beats on 24h-Holter monitoring), which enabled the definitive diagnosis of ACM.^8^

Nevertheless, as the modified TFC lacked sensitivity in the diagnosis of ACM with isolated or predominant LV involvement, Corrado et al. recently presented an International Expert Consensus document proposing the “Padua criteria”, which constitutes an upgrade of the diagnostic criteria of ACM aiming for the diagnosis of the entire spectrum of the phenotypic variants of ACM.^10^

In this recent consensus, new criteria have been added that reflect LV involvement, namely: (i) LV systolic dysfunction has been proposed as a minor criterion for diagnosing “biventricular” or “dominant-left” disease variants; (ii) LV myocardial LGE/fibrosis in the form of a stretch mark (or band) pattern affecting ≥1 segment of the LV free wall, septum, or both has been proposed as a major criterion; (iii) Repolarization abnormalities with inverted T waves in left precordial leads (V4-V6) (in the absence of complete LBBB) has been proposed as a minor criterion; (iv) Depolarization abnormalities with low QRS voltages in the limb leads (in the absence of obesity, emphysema, or pericardial effusion) has been proposed as a minor criterion, based on the notion that the decrease of LV myocardial mass by fibro-fatty replacement may lead to low QRS voltages; v) Frequent ventricular extrasystoles (>500 per 24 h), non-sustained or sustained ventricular tachycardia with an RBBB morphology (excluding the fascicular pattern) has been proposed as a minor criterion; and (vi) Demonstration of a pathogenic mutation in ACM-related genes has been considered a necessary criterion for the diagnosis in patients with left-dominant ACM and no clinically detectable RV involvement, because it is the most specific finding linking the LV phenotypic features to ACM.^10^

Indeed, all the aforementioned criteria are fulfilled in the index case of our report (except changes in the MRI because it was not performed during follow-up), thereby confirming the diagnosis of left-dominant ACM.

DCM is particularly difficult to distinguish from non-classic forms of ACM. These two entities can significantly overlap, which may result in a mislabeling of the diagnosis, as it probably occurred in our index case. Desmosomal gene mutations are relatively common in patients with a clinical diagnosis of DCM, and *DSP* mutations are found in 3% of patients with DCM.^11^

Left-dominant ACM may present over a wide range of ages typically with palpitations and impaired consciousness. Ventricular arrhythmia (VA) with RBBB morphology is characteristic and often out of proportion to the degree of LV dysfunction.

Palpitations, (pre)syncope, and VA are present at an early stage of ACM, often in the absence of gross structural abnormalities, as observed in both the index case and his son.

Genotype/phenotype studies have suggested that *DSP* mutations are associated with a severe phenotype with a higher risk of VA and SCD, and a high level of LV involvement, particularly in patients with truncating mutations, as observed in our patients. In addition, in line with our case, *DSP* mutations may be associated with T wave inversion in leads V4 to V6.^12^

In our case, the variant p.Trp360* was found in the *DSP* gene. Although it has never been described in the literature or genetic databases, this mutation results in a truncated protein, which may relate to a more aggressive phenotype, as seen in our family. Furthermore, pedigree analysis showed a positive congregation pattern, as the *DSP* mutation was found only in affected patients, but not in negative-phenotype patients, including the older ones.

This case shows the importance of *post-mortem* genetic study in patients with DCM/ACM phenotype, who suffered SCD before the genetic testing was performed.
